# A Dataset of Medical Questions Paired with Automatically Generated Answers and Evidence-supported References

**DOI:** 10.1038/s41597-025-05233-z

**Published:** 2025-06-19

**Authors:** Deepak Gupta, Davis Bartels, Dina Demner-Fushman

**Affiliations:** National Library of Medicine, National Institutes of Health, HHS, Bethesda, MD USA

**Keywords:** Information technology, Scientific data

## Abstract

New Large Language Models (LLM)-based approaches to medical Question Answering show unprecedented improvements in the fluency, grammaticality, and other qualities of the generated answers. However, the systems occasionally produce coherent, topically relevant, and plausible answers that are not based on facts and may be misleading and even harmful. New types of datasets are needed to evaluate the truthfulness of generated answers and develop reliable approaches for detecting answers that are not supported by evidence. The MedAESQA (Medical Attributable and Evidence Supported Question Answering) dataset presented in this work is designed for developing, fine-tuning, and evaluating language generation models for their ability to attribute or support the stated facts by linking the statements to the relevant passages of reliable sources. The dataset comprises 40 naturally occurring aggregated deidentified questions. Each question has 30 human and LLM-generated answers in which each statement is linked to a scientific abstract that supports it. The dataset provides manual judgments on the accuracy of the statements and the relevancy of the scientific papers.

## Background & Summary

The unprecedented improvements in the quality of the answers to medical questions generated by AI models are enabled by datasets comprised of question-answer pairs, such as MultiMedQA^1^. These traditional datasets were generated on the assumption that having pairs of questions and ideal answers along with the sets of relevant documents, such as PubMed abstracts, is sufficient to train and test the answer generation systems^2^. This assumption held for the answers that were traditionally extracted from the relevant documents. With the advent of Large Language Models (LLMs) capable of generating answers using solely their internal representations of the training data, in so-called zero-shot settings, it became clear that the coherent, grammatically perfect, and topically relevant answers may not necessarily be factual. Studies on the evaluation of LLMs’ abilities to support (ground) generated statements with verifiable evidence from reliable sources have shown that the models may provide harmful answers^1^, perform significantly worse on lay-user generated questions, and often fail to reference relevant sources^3^. This can pose a risk to public health^4^. Unsupported statements are, therefore, a major barrier to using LLMs in any applications that may affect health. Methods for grounding generated statements in reliable sources along with practical evaluation approaches are needed to overcome this barrier. To support these goals, we have developed MedAESQA (Medical attributable and evidence supported question answering) a publicly available dataset of naturally occurring health-related questions asked by the general population paired with sets of human and AI-generated answers. Each answer statement in the dataset is required to be supported by evidence, and the evidence and the documents containing the evidence are judged for accuracy and support. The dataset is designed to be used for developing, fine-tuning, and evaluating language generation models in several approaches that address the model’s ability to attribute or support the stated facts by linking the statements to the relevant passages of reliable sources. The approaches, Retrieval Augmented Generation (RAG)^5^ and retrofit attribution^6^, provide sources to the models to guide answer generation or to find support and post-edit the generated output, respectively. Additionally, approaches may interleave retrieval and generation tasks^7^. The non-medical Question-Answering datasets that were used to support attribution include the Natural Questions dataset^8^. The questions in this dataset consist of real anonymized aggregated queries seeking factual information using the Google search engine. The answers consist of a Wikipedia page, a bounding box on this page (effectively, a summary of the page), called the long answer, and the short answer, such as one or more named entities mentioned in the Wikipedia article, yes/no, or NULL, if the page does not answer the question.

**Fig. 1:**
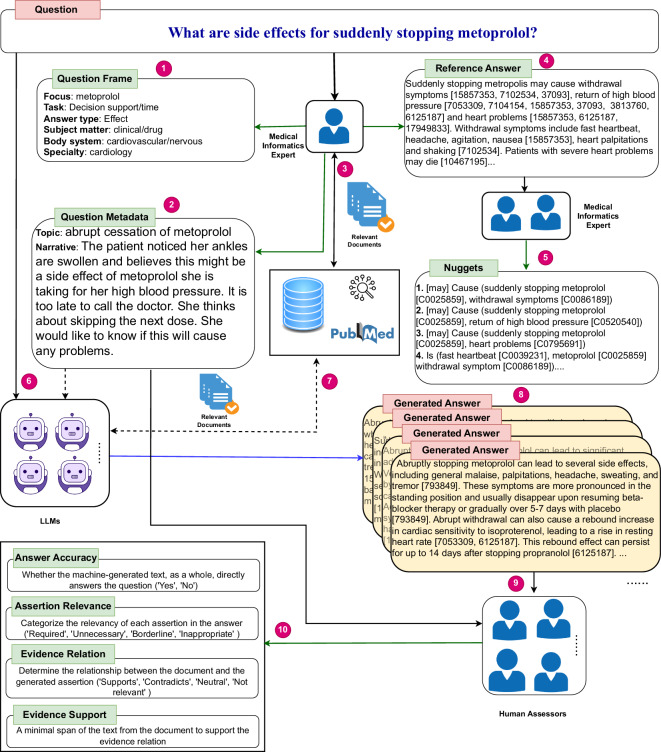
The schematic workflow of the MedAESQA dataset creation. The dataset creation starts with annotating the question frame ➊ and question metadata, which are the topic and narrative ➋. Thereafter, the medical information expert interacts with PubMed ➌ to retrieve the relevant PubMed articles and generates the reference answer ➍ with the appropriate references for each assertion in the reference answer. In the next step, two assessors formulated the nuggets ➎ from the reference answers. The question and metadata were given to the LLMs ➏, and the LLMs interact (optionally) with the PubMed collections and retrieve the relevant documents ➐, which they used to generate the answers with appropriate references. Once the answers are generated ➑, human assessors read the answers ➒, verify the references, and provide the multi-axis assessments ➓. The dotted arrow shows the optional interaction. The human assessors provided information is shown with a green arrow, while machine-generated answers are shown with a blue arrow.

In the medical domain, some datasets can be adapted to train models to support attribution. For example, the BioASQ data^9^ contains factoid, yes/no, list, and summary questions formulated by biomedical experts. The questions are linked to sets of biomedical terms (concepts) related to the question and a set of research articles that are sufficient to answer the question. Text snippets containing one or more sentences that answer the question fully or partially are marked in the articles by the experts. The dataset is primarily focused on drug-target-disease relations for medical investigations. The MEDIQA-AnS dataset^10^ contains consumer-health questions, the full text from reliable web pages, extracted passages from the full text, and manually created summaries. In general, an attribution verification dataset must contain a question and at least one answer in which each statement of a fact required to answer the question is annotated and linked to a corresponding statement in an evidence source that supports or contradicts the fact stated in the answer. While the above datasets may be retrofitted to adhere to this format, to the best of our knowledge, we present the first medical question answering dataset specifically generated to test attribution to identified sources when assessing the output of natural language generation models. The distinct characteristics of the MedAESQA dataset are as follows: 1) the questions are naturally occurring popular questions submitted by the public to the National Library of Medicine, 2) the questions are annotated with the main concept of interest and with the user’s intent, e.g., to learn a fact or to support a clinical decision; 3) each question has a manually generated answer in which each sentence is linked to a PubMed abstract; 4) each question has 30 answers automatically generated by large language models. Each statement in the automatically generated answers is manually judged as required, unnecessary, or inappropriate. Each PubMed abstract provided by the models to support the specific statements is also manually judged as supporting, contradicting, or topically relevant or not to the answer. Finally, in each document that supports or contradicts the answer statements, a specific passage of text is annotated as evidence that supports the judgment. Figure 1 provides the workflow of the dataset creation and an example of a data entry.

## Methods

### Question Formulation

The MedAESQA questions are developed using information requests submitted by self-identified non-clinicians to the MedlinePlus^11^ service provided by the National Library of Medicine. We chose the previously unseen, most popular, forty questions asked by MedlinePlus users. Each question also includes medical informatics expert-coded *topic* and *narrative* to support the efficient retrieval of the relevant documents. The *topic* signifies the key subject (focus) of the question, whereas *narrative* provides the context and background information on the question. Additionally, we provide an expert-coded structured representation of the information in the question, which we call question frame^12^. Frame representation resembles a predicate-argument structure where a predicate is connected to its arguments and their semantic roles, such as THEME and AGENT^13^. A question frame includes a trigger for the question type, one or more THEME arguments, and optional semantic roles, all tied to their text mentions. In a question frame, *question focus* is the main theme of the question, which describes the key entities of the question, and the *question type* signifies the aspect of interest about the question focus (expected answer type). To characterize the disorders, user intents, body systems and anatomical structures, and clinical specialties encompassed by the collection, we also label each question with its ‘*Subject Matter*’, i.e., the broad medical area, such as genetics, or focus on clinical drugs; ‘*Body System*’, and ‘*Specialty*’, i.e., a clinical expert best suited to answer the question, such as cardiology or endocrinology. These labels show that the MedAESQA dataset contains questions that look for a variety of answers: *treatment*, *effect*, *etiology*, etc. In our further fine-grained analysis on Body System on MedAESQA questions, we found that questions cover almost all the body systems, starting from ‘*immune*’ to ‘*vision*’. We also analyzed the Clinical Specialty of the medical specialist that would typically address or manage the issue, and we observed that MedAESQA questions cover an array of diverse specialties. We have provided the distribution of the question frame (Task, Answer type, and Subject Matter) and list of all Body Systems and Specialties of MedAESQA questions in Table 1 and 2, respectively.

**Table 1:** Distribution of the different question frame categories in the MedAESQA dataset.

Question Frame Category	Count	Type
Task	15	Decision support
	25	Information seeking
Answer type	10	Treatment
	5	Etiology
	18	Effect
	2	Indications
	1	Prognosis
	1	Diagnosis
	3	Definition
Subject matter	4	Genetics
	22	Clinical / Problem
	8	Clinical / Drug
	6	Clinical / Intervention

**Table 2:** Body Systems and Clinical Specialties covered in questions present in the MedAESQA dataset.

**Body System**	cancer, cardiovascular, cardiovascular/nervous, digestive, endocrine,endocrine/metabolism, full body, immune, infectious, integumentary, mental, metabolism, musculoskeletal, nervous, reproductive, reproductive/endocrine, reproductive/urinary, respiratory, vision
**Specialty**	cardiology, cardiology/pulmonology, clinical, genetics, dermatology, endocrinology, gastroenterology, GP, GP/cardiology, GP/rheumatology /sport medicine, hepatology, immunology, neurology, neurology/clinical genetics, ob./gyn, oncology, ophthalmology, orthopedic, surgery, psychiatry, pulmonology, pulmonology/ENT/cardiology, surgery, urology

### Expert-curated Answers

Following the work of Attal *et al*.^14^, a medical information expert utilizes the question focus and answer type to query PubMed^15^ and retrieve articles that could potentially provide the answer to the question. In the next step of answer formulation, the expert reads the titles and abstracts of relevant articles and formulates an answer sentence by considering one or more abstracts. For each answer sentence, the expert also includes the appropriate PMIDs to provide evidence for the assertions stated in the answer sentence. By following the aforementioned strategy, an answer (with multiple sentences) is formulated in such a way that it remains complete, accurate, coherent, and evidence-supported with appropriate PubMed identifiers (PMIDs) for each assertion.

### Expert-curated Nuggets

Additionally, we provide manually generated information nuggets for factual evaluation. An information nugget can be used by an assessor to make a binary decision as to whether the fact represented by said nugget is contained in a response^16^. An assessor may determine nuggets to be required for an answer and may match nuggets to the sentences that contain them. This allows for a finer level of granularity in the evaluation and the assessment of an atomic fact rather than a sentence as a whole. Nuggets were generated from the 40 expert-curated answers in the MedAESQA dataset, where exactly one nugget was generated for every fact contained in an answer. We used a Predicate (subject, object) form to capture the information nuggets. Each medical concept in a nugget is associated with a Concept Unique Identifier (CUI) from the Unified Medical Language System (UMLS)^17^. These CUIs were identified by manually searching the UMLS Metathesaurus Browser for the closest match. Some facts required more complex nugget structure including, but not limited to, “if, then” clauses and comparisons. An attempt was made to normalize language across answers with common predicates and formatting (e.g. Treat (treatment, condition) or Prevent (method of prevention, condition)), while retaining information from the original sentence as much as possible. Each nugget was reviewed by at least two reviewers.

### Machine-generated Answers

To generate answers with appropriate references, we organized a community evaluation^18^ at the 2024 Text Retrieval Conference (TREC). The participants proposed their approaches for generating answers. Analyzing the participants’ approaches, we outline a framework that takes an input question along with additional metadata (topic and narrative) and provides the generated answer as output.

The detailed steps include:

1. **Query Formulation and Expansion:** Given a topic, question, and narrative, a query is formulated to search PubMed articles to obtain the relevant documents from PubMed collection. The query can be formulated by considering either the topic, question, narrative, or any combination of these metadata available with each question. To improve the retrieval process query expansion can also be applied where a query is expanded or transformed with additional terms or phrases that are semantically related or contextually relevant.
2. **Document Retrieval**: To retrieve the relevant documents, the 2024 annual baseline snapshot of Medline/PubMed, which goes approximately through the end of 2023 was used. We provided a pre-processed set of 20, 727, 695 PMIDs representing the abstracts in the 2023 snapshot. The approaches have primarily used lexical retrieval (BM25^19^) to retrieve the top-k relevant documents for each question by utilizing the index built upon the title and abstract of the PubMed collection. The approaches also experimented with extracting the relevant snippets from the documents and considered the snippets as the relevant passages for the next stage of the framework.
3. **Document Reranking:** The ranking of the documents/snippets is an important step to further improve the ranking of documents retrieved in the first stage of the retrieval system. The goal is to reorder the retrieved documents to present the most relevant and high-quality results at the top of the list. Multiple re-rankers were utilized to re-rank the documents/snippets: pointwise (monoT5^20^, TAS-B^21^, ANCE^22^), pairwise (duoT5), and listwise (RankGPT^23^) approaches to rerank the documents/snippets.
4. **Answer Generation:** The reranked documents along with the corresponding question were used to generate an answer to the question. Various open (Mistral-7B^24^, Llama3.1^25^) and closed-sourced (gemini-1.5-flash-001^26^, gpt4o-mini, GPT-4o^27^) LLMs were utilized to generate the answers, additionally, LLMs were instructed to cite the appropriate PMIDs of the ranked documents while stating the fact in the answer.
5. **Post-hoc Citations (Optional):** This is an optional alternative step in our framework, where an answer is generated first without referencing any documents, and in the post-hoc stage, each sentence is required to cite supporting documents. LLMs were employed to provide appropriate citations for each sentence from the reranked list of the documents.

### Human Judgment on Answers

We evaluated two different aspects of the answers: **(a)** reference attribution and **(b)** the quality and factuality of the answers. The former aims to judge the support the referred documents provide for an assertion generated by the machine and the latter focuses on evaluating the answer to a clinical question asked by clinicians to answer health-related questions asked by their patients. We envisioned that clinicians would review each answer and subsequently explain it in plain language. The evaluation was conducted by clinicians employed by Centaur Labs^28^ in a crowdsourcing manner. Each final judgment is a consensus on the majority vote of at least three annotators. We also computed the inter-annotator agreement score, which is defined as the percentage of annotators who assigned the majority label to the total number of annotators. We have provided the details of inter-annotator agreement scores on different annotation tasks in the creation of the MedAESQA dataset in the Table 3.

**Table 3:** Detailed agreement scores on different annotation tasks in the creation of the MedAESQA dataset.

Annotation Task	Agreement	Labels
Answer Accuracy	90.37	Yes
	76.06	No
Assertion Relevance	72.39	Required
	47.15	Borderline
	48.30	Unnecessary
	50.58	Inappropriate
Evidence Relation	82.09	Supporting
	67.67	Contradicting
	60.14	Neutral
	100	Not Relevant

We follow a two-step judgment on machine-generated answers:

- **Step 1: Evaluating Answer Alignment with Questions and Answer Quality and Completeness:** We begin the evaluation by assessing whether the machine-generated text, as a whole, directly answers the question. In the next step, we examined the relevance of each assertion in the answer sentences to the question. Toward this, we categorized each assertion in the generated answer using one of the following four labels:
  - **Required**: Given assertion is necessary to have in the generated answer for completeness of the answer.
  - **Unnecessary**: Given assertion is not required to be included in the generated answer. An assertion can be categorized as unnecessary for multiple reasons including **(a)** it provides general information on the topic, **(b)** it recommends seeing a doctor, while the task states the patient has already contacted the provider, or the provider is asking the question.
  - **Borderline**: An assertion can be marked borderline, if it is relevant, possibly even -“good to know”,- but not required to be part of the answer. For example, if the question is about the most commonly used treatments, information about treatments in the early stages of clinical trials is not necessary.
  - **Inappropriate**: If an assertion may harm the patient if followed, it is marked as inappropriate. E.g., if, according to the answer, physical therapy reduces the pain level, but the patient experiences more pain due to hip mobilization, the patient may start doubting they are receiving adequate treatment.
  We have provided examples of the borderline and unnecessary answer sentences for some healthcare questions in Table 4.

**Table 4:** Sample examples showing the assertion relevance category for *borderline* and *unnecessary* labels.

Relevance	Question	Assertion
borderline	Are there ways to prevent sleep apnea or treat it naturally?	It is essential to note that while these natural methods can be helpful, they may not be as effective as continuous positive airway pressure (CPAP) therapy, which is the gold standard treatment for sleep apnea.
borderline	What does CAD without angina mean?	Therefore, it is crucial for patients diagnosed with CAD without angina to be monitored closely for potential heart issues.
borderline	What are the short and long term effects of surgery and its complexity for women with ovarian cyst?	Laparoscopic surgery is generally considered a safe and effective treatment for ovarian cysts, with a lower risk of complications and a faster recovery time compared to open surgery.
borderline	what is the best test to rule out a lumbar disc herniation?	This system can detect all visible herniated discs regardless of their location.
unnecessary	Are there ways to prevent sleep apnea or treat it naturally?	There is no specific context provided regarding natural prevention or treatment methods for sleep apnea.
unnecessary	What are the side effects of using formoterol?	However, these effects are generally not clinically significant.
unnecessary	can i use Sudafed after using Afrin for nasal congestion?	Always consider individual patient factors and consult clinical guidelines or a healthcare provider if uncertain.
unnecessary	When should I treat high blood pressure?	This recommendation is strongly supported by the literature.
unnecessary	What are the effectiveness of physical therapy interventions in reducing pain in patients with lumbar disc herniation?	The mathematical expression ability of the logistic regression model for patients with lumbar disc herniation undergoing physical therapy was sufficient.
- **Step 2: Evaluating Answer Alignment with Evidence Support:** In the second step, we evaluated the referenced document(s) for each generated answer sentence to determine the relationship between the document and the generated assertion, if any. Each cited document was labeled with one of four possible relationships to the answer sentence: *‘Supports’*, *‘Contradicts’*, *‘Neutral’*, or *‘Not Relevant’*. Additionally, the experts also provided a passage from the referenced document to support their assessment of the evidence relation.
  - **Supports**: A relation between the referenced document and the answer sentence is marked as *support*, if there is at least one sentence in the referenced document that supports/agrees with the assertion made in the answer sentence, e.g.: *“opioids were the mainstay of perioperative pain control”*. In addition, no other sentence in the document contradicts the statement.
  - **Contradicts**: A relation between the referenced document and the answer sentence is marked as *contradicts* if there is at least one sentence in the referenced document that disagrees with the assertion or states its opposite, e.g.: *“Increasing pain levels after the first week postoperatively, for 3 days, are most likely to be caused by the change to more extensive mobilization and physiotherapy in the rehabilitation unit.”* (The answer in this case stated that the pain decreases steadily after the surgery.)
  - **Neutral**: The referenced document is marked *neutral*, if it is topically relevant, but lacks any information to validate or invalidate the assertion made in the answer sentence.
  - **Not relevant**: The referenced document is considered *Not Relevant* if the referenced document is not relevant to the sentence.

## Data Records

**Fig. 2:**
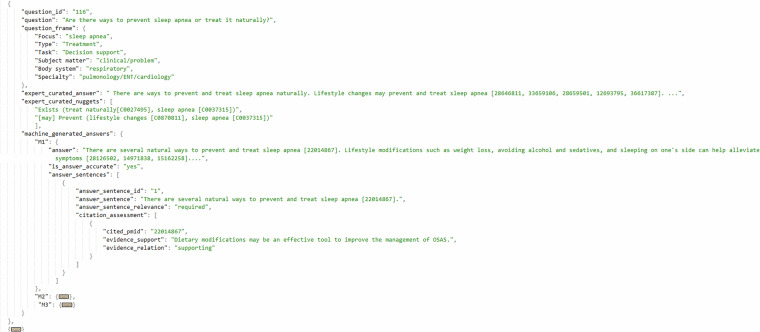
An example of a JSON tree for one of the data objects in the MedAESQA dataset. The illustration shows answers for only three machine-generation approaches M1, M2, and M3, however, the dataset has answers from 30 machine-generation approaches (M1 to M30) along with the citation assessment for each answer sentence in the generated answers. The answers and nuggets are truncated in the example.

We have archived MedAESQA^29^ data records with Open Science Framework (OSF), available at 10.17605/OSF.IO/ydbzq. The OSF link contains a directory called MedAESQA. The MedAESQA directory contains the entire dataset in a JSON file, which lists data items. The README file also contains detailed information about each field in the dataset, including sample code to process the data. Each item in the JSON file contains the relevant key (metadata name) and value (metadata information) pairs, which are *question_id*, *question*, *question_frame*, *expert_curated_answer*, *expert_curated_nuggets* and *machine_generated_answers*. The detailed statistics of the MedAESQA dataset are shown in Table 5. Figure 2, shows the JSON tree to visualize the data samples.

**Table 5:** Detailed MedAESQA dataset statistics for questions, expert-curated answers, machine-generated answers, citations, and evidence.

Statistics	Question	Expert-curated Answer	Machine-generated Answer	Citations in Expert-curated Answer	Citations in Machine-generated Answer	Evidence
**Sum**	40	40	1200	316	8111	7651
**Min**	5	43	8	3	0	1
**Max**	23	222	201	10	31	322
**Mean**	10.6	98.65	101.15	7.925	6.67	33.57
**Median**	9.5	93.0	110	8.0	6.0	29

The statistics for questions, answers, and evidence denote the number of tokens they have after performing tokenization with the NLTK tokenizer.

## Technical Validation

To generate multiple answers and validate the MedAESQA dataset, we organized a community evaluation^18^ at the 2024 Text Retrieval Conference (TREC), in which participants were provided with the questions and PubMed collection and asked to generate the answers in which the appropriate PMIDs supported each assertion (equated to sentences in this evaluation). We acknowledge that in this edition of the TREC evaluation, which resulted in the MedAESQA dataset, we limit ourselves to the title and abstract of the PubMed document, which may inhibit real-world usability of supporting the assertions. In the future, we plan to use the full-text PubMed document to assess the validity of the assertion. Participants were asked to generate the answer sentence that has to be supported by up to three attributions (cited references), with a maximum of 30 documents allowed per answer. Documents had to be cited in the answers using PMIDs enclosed in square brackets, as illustrated in Figure 1. For each question, we received thirty answers generated using different approaches. All the answer-generation approaches are depicted in the Appendix. The different strategies used by the participants offer diverse answers. Furthermore, each answer as a whole and the assertions with corresponding citations are manually evaluated by the experts, which provides the level of correctness of the machine-generated answers. We next describe the metrics we used to validate the answer quality, associations between the citations and assertions, and relevance of the cited documents.

### Benchmarking Metrics

We conducted a comprehensive evaluation of machine-generated answers across multiple levels and dimensions.

- **Answer Quality**: We evaluate the quality of the generated answers considering multiple perspectives: accuracy, precision, recall, and redundancy. The details of the metrics are as follows:
  - **Answer Accuracy** evaluates the accuracy of the generated answer using human-provided judgment. It measures how many of the answers to the total of 40 questions were deemed acceptable (judged as answering the question at least partially) for each answer generation approach.
    1 $$\,{\rm{Accuracy}}=\frac{{\rm{Number\; of\; Acceptable\; Answers}}}{{\rm{Total\; Number\; of\; Questions}}}$$
  - **Answer Completeness (Recall)** evaluates the extent to which a given answer covers the facts (aspects) deemed required by the assessors. The required aspects are aggregated across all system-generated answers. To identify answer aspects, we cluster the answer sentences using sentence embeddings generated by the SentenceTransformer model (sentence-transformers/all-mpnet-base-v2) and the SimCSE model (princeton-nlp/sup-simcse-roberta-large). We set up multiple evaluation levels for computing recall. In a strict evaluation, only sentences judged required and supported by evidence were considered for grouping. For a lenient evaluation, all sentences judged required were considered. For a relaxed evaluation, the borderline sentences were considered in addition to the required sentences. The number of aspects for the automated grouping is set to 10 using K-means clustering.
    2 $$\,{\rm{Completeness}}=\frac{{\rm{Number\; of\; Distinct\; Clusters\; Containing\; Sentences\; from\; Answer}}}{{\rm{Number\; of\; Clusters}}}$$
  - **Answer Precision** assesses the proportion of the assertions that were judged required or acceptable in the answer.
    3 $$\,{\rm{Precision}}=\frac{{\rm{Number\; of\; Generated\; Required\; Sentences}}}{{\rm{Total\; Number\; of\; Generated\; Sentences}}}$$
  - **Redundancy Score** quantifies *unnecessary* answer sentences and penalizes a system for generating unnecessary sentences. This score measures the informativeness of the generated answers as a proportion of generated *unnecessary* answer sentences among all generated answer sentences.
    4 $$\,{\rm{Redundancy\; Score}}=\frac{{\rm{Number\; of\; Generated\; Unnecessary\; Sentences}}}{{\rm{Total\; Number\; of\; Generated\; Sentences}}}$$
  - **Irrelevancy Score** quantifies *inappropriate*/*potentially harmful* answer sentences and penalizes a system for generating these sentences. The score measures the potential of the generated answer to mislead the reader.
    5 $$\,{\rm{Irrelevancy\; Score}}=\frac{{\rm{Number\; of\; Generated\; Inappropriate\; Sentences}}}{{\rm{Total\; Number\; of\; Generated\; Sentences}}}$$
- **Citation Quality:** A system-generated answer statement may be supported or contradicted by the documents provided as references. It is also possible that answer sentences may not include any references or may include references that are only topically relevant or irrelevant. The following metrics are designed to assess the quality of these references:
  - **Citation Coverage** measures how well the required and borderline generated answer sentences are backed by the appropriate (judged as supports) citations.
    6 $$\,{\rm{Citation\; Coverage}}=\frac{{\rm{Number\; of\; Systems\; Generated\; Answer\; Sentences\; with\; One\; or\; More\; Supportive\; Citation}}}{{\rm{Total\; Number\; of\; Generated\; Answer\; Sentences}}}$$
  - **Citation Support Rate** assesses the proportion of the citations provided by a system that were judged by the experts as supporting the corresponding statement in the generated answer.
    7 $$\,{\rm{Citation\; Support\; Rate}}=\frac{{\rm{Number\; of\; Supports\; Citations}}}{{\rm{Total\; Number\; of\; Citations}}}$$
  - **Citation Contradiction Rate** assesses the proportion of the citations provided by a system that were judged by the experts as contradicting the corresponding statement in the generated answer. In a fact-verification task, this measure can indicate how effectively a system identifies contradictory evidence.
    8 $$\,{\rm{Citation\; Contradict\; Rate}}=\frac{{\rm{Number\; of\; Contradict\; Citations}}}{{\rm{Total\; Number\; of\; Citations}}}$$
- **Document Relevancy:** By pooling all documents judged relevant to a given topic, we compute standard recall and precision. The set of relevant documents includes documents judged as supporting, contradicting, or neutral.
  9 $$\,{\rm{Recall}}=\frac{{\rm{Number\; of\; relevant\; retrieved\; documents}}}{{\rm{all\; relevant\; documents}}}$$
  10 $$\,{\rm{Precision}}=\frac{{\rm{Number\; of\; relevant\; retrieved\; documents}}}{{\rm{Number\; of\; references\; provided}}}$$

### MedAESQA Dataset Analysis

**Fig. 3:**
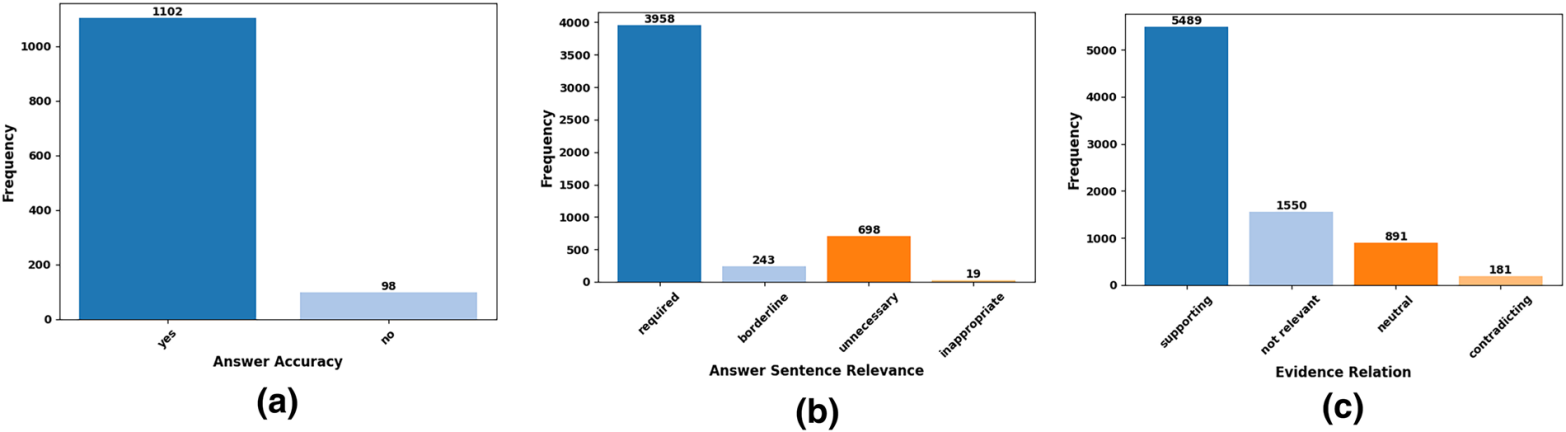
Distribution of the assessed labels for answer accuracy **(a)**, answer sentence relevance **(b)**, and evidence relation **(c)** in MedAESQA dataset.

We performed a detailed analysis of the developed MedAESQA dataset by calculating dataset statistics at various levels of granularity. We found 1,108 out of 1,200 machine-generated answers were deemed acceptable by experts. We also analyzed the answer sentence relevancy assessed by the experts and found that 3,958 answer sentences were judged *required* out of 5,162 generated answer sentences. Similarly, in our analysis of the evidence relation, we found 5489 references out of 8111 *supported* the assertion made in the generated answers. The detailed analysis of different categories of answer sentence relevancy and evidence relation is presented in Fig. 3.

The MedAESQA dataset comprises 40 questions along with their expert-curated and machine-generated answers. Each question is associated with 30 machine-generated answers, resulting in a total of 1,240 answers. For each machine-generated answer, a sentence-level assessment is conducted to evaluate answer accuracy, sentence relevance, and evidence relation. The dataset includes a total of 5,162 answer sentences. In the expert-curated answers, we identified 316 references, with a minimum of 3 and a maximum of 10 references per answer. On the other hand, the machine-generated answers yielded a minimum of 0 and a maximum of 31 references per answer. The MedAESQA dataset also contains a total of 7,651 human-curated evidence excerpts from referring documents to support the assessed evidence relations. A detailed analysis of the MedAESQA dataset is provided in Table 5.

### Benchmarking Evaluation

We evaluated the performance of methods used to generate answers in the created MedAESQA dataset on various evaluation metrics. The detailed results are presented in Tables 6, 7 and 8. On the answer accuracy metric, 26 out of 30 methods achieved more than 92% accuracy with a maximum of 100% (11 methods) and a minimum of 92.5% (2 methods). More than one-third of the methods (11 out of 30) achieved perfect accuracy which shows the acceptable quality of the generated answers. For precision of the answer, method M17 achieved the best performance with a precision score of 90.23. The precision score for 17 out of 30 methods was in the range of 73.54 to 85.54 and for 3 out of 30 methods, the precision score was in the range of 85.54 to 90.23. The redundancy scores were high (>15%) for only five methods. Method M4 recorded the lowest redundancy score of 4.04% with a precision of 79.08. Harmfulness is a key metric that aims to assess the tendency of the system to generate harmful sentences. We found that 19 of 30 methods achieved a perfect harmfulness score of 0%. The remaining methods also perform well and seem cautious while generating the answers as the highest harmfulness score we recorded was only 1.88. Nevertheless, we believe the harmful sentences are a good source for training and testing approaches for identifying and mitigating harmful answers.

**Table 6:** Performance comparison of the multiple machine-generated answers regarding answer quality focusing on accuracy, precision, redundancy, and harmfulness metrics.

Method	Acceptable Answers	Accuracy	Precision	Redundancy	Harmfulness
M1	39	97.5	86.29	5.29	0
M2	40	100	82.98	13.23	0
M3	38	95	80.08	8.29	1.62
M4	37	92.5	79.08	4.04	0
M5	23	57.5	49.54	6.83	0
M6	40	100	81.1	13.25	0
M7	33	82.5	63.54	16.46	0
M8	38	95	71.15	19.55	0
M9	39	97.5	73.93	17.42	0.77
M10	40	100	82.29	13.66	0.36
M11	39	97.5	83.75	8.38	0.5
M12	38	95	84.5	6.12	0
M13	32	80	58.62	16.19	0
M14	36	90	72.32	14.07	0
M15	26	65	54.17	8.75	0
M16	40	100	78.92	16.18	0.42
M17	40	100	90.23	6.77	0.5
M18	37	92.5	77.46	10.21	0.62
M19	40	100	81.92	14.36	0.62
M20	38	95	82.88	5.42	0
M21	36	90	79.62	4.29	0.62
M22	26	65	55.33	7.37	0
M23	30	75	57.92	12.5	0
M24	40	100	81.29	12.85	1.2
M25	40	100	79.79	16.46	0
M26	40	100	79.35	13.26	0
M27	40	100	78.48	17.42	0
M28	40	100	70.83	21.92	1.88
M29	39	97.5	88.17	4.25	0
M30	38	95	62.5	27.92	0

**Table 7:** Performance comparison of the multiple machine-generated answers regarding answer quality focusing on completeness (recall) metric using two different approaches to form the cluster: Sentence Transformer and SimCSE.

Method	Sentence Transformer			SimCSE		
	Recall (S+R)	Recall (R)	Recall (R+B)	Recall (S+R)	Recall (R)	Recall (R+B)
M1	28.75	30.25	31.5	29.25	31	31.25
M2	28.75	38	39.75	29.5	37.75	39.5
M3	25.25	30	31	25.5	29.75	30.25
M4	26.5	27.5	31	27	28.5	30.5
M5	5.5	7.25	7.75	5.5	7.5	7.75
M6	30.75	38.25	41.5	30.75	37.75	40.5
M7	7.25	14.5	15.25	7.25	14.25	15
M8	22.75	31.75	32.75	24.25	31.5	32.75
M9	26	33.5	31.75	26.25	32.5	34.5
M10	24	37.5	38.25	25.25	35.75	36.25
M11	29	29.75	31.75	29.5	31	31.25
M12	24.5	27.25	28.5	25.25	28	28
M13	20.25	29	30.5	23.25	29.25	33.5
M14	23.25	35	35.75	23.75	32.75	33.25
M15	4.75	8	9	4.5	8	8.5
M16	32.5	36.75	36.75	33	37.25	40
M17	25.5	28.75	29	25.25	28.75	29.25
M18	25.75	28.75	29.75	26.25	29.75	30
M19	32.75	37	39.25	32.5	40.25	41
M20	27.75	29.75	32	28.75	30.75	31.25
M21	26	26.75	28.25	24	27	29.25
M22	3.5	8	8.5	3.75	8.25	9.25
M23	6.25	8.5	10	6	9	9.5
M24	40.25	43.5	42.75	40.75	44.5	45.75
M25	0	20.75	20.25	0	20.75	21.5
M26	31.5	35.75	39	31.25	37.5	41.5
M27	0	39.75	40.25	0	38.75	41
M28	2	25	26.25	2	24.75	26
M29	26.75	31.25	32.75	27.75	31.75	32.5
M30	7.75	16.5	17.5	7.25	16.25	17.75

The abbreviations are defined as follows: (**S+R**)—only answer sentences deemed required and supported by evidence were included for clustering; (**R**)—answer sentences identified as required were included for clustering; (**R+B**)—answer sentences deemed as either required or borderline were included for clustering.

**Table 8:** Performance comparison of the multiple machine-generated answers regarding citation quality and document relevance on their respective metrics.

Method	Citation Coverage	Citation Support Rate	Citation Contradict Rate	Document Recall	Document Precision
M1	91.92	72.34	2.84	9.63	83.57
M2	64.67	66.08	0	7.96	70.93
M3	77.62	61.56	3.03	8.05	76.54
M4	86.46	70.27	3.45	11.37	80.55
M5	42.75	41.25	3.75	0.86	41.25
M6	75.52	75.76	3.86	11.83	89.14
M7	43.12	70.42	2.5	2.33	77.5
M8	64.32	55.14	1.23	8.1	59.71
M9	68.66	61.08	0.15	7.66	67.24
M10	58.61	55.32	1.33	6.85	64.76
M11	90.54	74.47	4.97	11.84	83.58
M12	83.38	67.77	1.29	9.27	75.67
M13	52.15	47.81	0.28	4.97	51.04
M14	56.55	62.33	0	7.05	65.68
M15	32.5	40	5	1.18	55
M16	76.98	73.54	2.39	13.01	87.02
M17	85.37	52.18	3.64	8.63	70.76
M18	81.88	61.29	3.14	10.5	74.94
M19	74.08	74.6	3.02	12.55	88.23
M20	86	68.77	4.7	11.49	79.57
M21	81.99	58.94	2.21	9.56	74.84
M22	31.04	36.25	0	0.75	36.25
M23	47.08	35.58	3.46	2.66	45.19
M24	85.32	57.77	1.91	23.77	74.98
M25	0	0	0	0	0
M26	78.33	77.88	1.82	11.62	86.87
M27	0	0	0	0	0
M28	6.38	13.75	0	0.77	29.58
M29	84.75	63.95	3.48	9.78	72.09
M30	33.75	40.75	3	2.64	52.92

To measure the answer completeness (recall), we followed a clustering approach where we clustered all the generated answers for a given question together to assess the distinct aspects that are covered in the machine-generated answers. We utilized two different answer sentence representation approaches Sentence Transformer and SimCSE. The comparative results under three different settings (S+R, R, and R+B) are demonstrated in Table 7. S+R, which considers only supported and required facts, is the most strict evaluation and R+B, which includes all required and borderline statements disregarding the support, is the most lenient evaluation. We found that method M24 (Sentence Transfomer) obtained the best recall scores with 40.25 and 42.75 on the S+R and R+B settings respectively. A similar trend is also observed while using SimCSE as the sentence representation. Some methods recorded recall scores of 0 under S+R settings because those methods did not generate the citations for the generated answers.

While analyzing the results of the citation quality of the different approaches to the machine-generated answers, we observed that the majority of the approaches (18) achieved citation coverage in the range of 62 to 93. Method M1 recorded the highest citation coverage of 91.92 and M28 (excluding M25 and M27 as these methods did not generate citations along with the answer) as the lowest citation coverage of 6.38. For the citation support rate (CSR) the method M26 obtained the highest score of 77.88. Citation contradiction rate (CCR) is another key metric to evaluate citation quality as it assesses how often a system is citing a contradictory document with an assertion. We found some of the best CSR score systems, M6 and M26 recorded CCR values of 3.86 and 1.82 which signify that the answer generation systems were good in citing the appropriate documents. We also analyzed the document recall and precision scores and observed that most of the systems yield low recall however the precision scores were moderate. Method M24 achieved the highest document recall of 23.77 with a precision of 74.98.

#### Expert-curated Answers vs. Machine-generated Answers

We also analyze how close the machine-generated answers are to the expert-curated answers. Towards this, we analyze answer-level and citation-level similarities between two different modes of the answer-curation. For the answer-level similarity, we computed the sentence similarly between the expert-curated and each machine-generated answer and reported the BLEU, BLEU-4, ROUGE-2, and ROUGE-L metrics. Since an answer can be stated in different way, n-gram similarities may not always be the best choice to measure the similarity, therefore, we also reported the semantic similarity by reporting the BERTScore between the the expert-curated and each machine-generated answer. The detailed results are shown in Table 9. On n-gram similarities-based metrics, we found that method M11 achieved the highest BLEU and ROUGE scores (0.0117 and 0.1845) and comparable BERTScore (0.8514). The method M20 records competitive scores to M11 on both n-gram-based evaluation (BLEU: 0.117, ROUGE: 0.1825) and semantic similarity (BERTScore: 0.8517) metrics. For citation-level similarity, we created a list of cited documents in the answer to a given question for expert citation and machine-generated citation. We considered the expert citation list as the ground truth citation and computed the true positives (generated citations that are also present in the expert citation), false positives (generated citations that are not present in the expert citation), and false negatives (expert citations that are not present in the generated citation). With these, we computed the citation precision, recall, and f-score and reported the performance in Table 9. We found that method M1 achieved the highest citation F-score of 13.63, however, method M11 and M20 (best on answer level similarity) also recorded the competitive F-Score of 13.00 and 12.46 respectively.

**Table 9:** Comparison of the different approaches of machine-generated answer to the expert-curated answer on answer level and citation level.

Method	BLEU	BLEU-4	ROUGE-2	ROUGE-L	BERTScore	Precision	Recall	F-Score
M1	0.05554	0.01176	0.07979	0.18227	0.85199	0.17152	0.11314	0.136344
M2	0.0433	0.00763	0.07305	0.16934	0.85336	0.07806	0.04531	0.057338
M3	0.05145	0.01124	0.07092	0.17236	0.8462	0.1026	0.05329	0.070146
M4	0.05334	0.01123	0.07793	0.17891	0.85082	0.14716	0.10548	0.122882
M5	0.01259	0.0023	0.04227	0.12463	0.8343	0.0375	0.00528	0.009257
M6	0.03682	0.00777	0.0633	0.15439	0.84595	0.12607	0.10843	0.116587
M7	0.02328	0.00356	0.05313	0.14594	0.84487	0.0375	0.0059	0.010196
M8	0.04651	0.00812	0.07188	0.1719	0.84964	0.07098	0.06082	0.065508
M9	0.05314	0.01213	0.07149	0.17378	0.85036	0.06868	0.03482	0.046211
M10	0.03811	0.00673	0.06317	0.1638	0.85069	0.06653	0.04208	0.051553
M11	0.05624	0.01177	0.08055	0.18457	0.85146	0.14097	0.12062	0.130003
M12	0.04739	0.00987	0.07082	0.178	0.84883	0.09607	0.06916	0.080424
M13	0.0454	0.00936	0.06847	0.16402	0.84543	0.03458	0.02201	0.026899
M14	0.03736	0.00533	0.06881	0.17233	0.85221	0.10268	0.05503	0.071657
M15	0.0079	0.00145	0.0438	0.12564	0.84	0	0	0
M16	0.03749	0.00629	0.07014	0.16769	0.84773	0.11595	0.10891	0.11232
M17	0.04158	0.00587	0.06793	0.17162	0.84869	0.13234	0.10082	0.114449
M18	0.04934	0.01	0.06661	0.16548	0.845	0.09605	0.07815	0.08618
M19	0.04396	0.00879	0.07187	0.167	0.84733	0.134	0.10531	0.117935
M20	0.05592	0.01177	0.07983	0.18254	0.85171	0.13295	0.11735	0.124664
M21	0.05451	0.01151	0.08123	0.17421	0.84874	0.06339	0.05631	0.059641
M22	0.01259	0.0023	0.04227	0.12463	0.8343	0.0125	0.0025	0.004167
M23	0.01632	0.00308	0.049	0.14014	0.84106	0.00833	0.00278	0.004169
M24	0.03261	0.00535	0.05721	0.15507	0.84394	0.06781	0.14099	0.091576
M25	0.0388	0.00661	0.07707	0.18306	0.85751	0	0	0
M26	0.03803	0.00777	0.05811	0.15567	0.8458	0.11603	0.09725	0.105813
M27	0.03598	0.00553	0.06613	0.16127	0.849	0	0	0
M28	0.03509	0.00471	0.06379	0.1685	0.84845	0	0	0
M29	0.05404	0.01164	0.07577	0.17963	0.84927	0.12656	0.07342	0.09293
M30	0.03206	0.00542	0.0625	0.15555	0.84637	0.05833	0.01632	0.025504

## Usage Notes

We have provided detailed instructions in the README file of the Open Science Framework repository (https://osf.io/ydbzq) describing how to process the MedAESQA datasets. The source code to evaluate the system performance can be found in the GitHub repository (https://github.com/deepaknlp/MedAESQA).

## Supplementary information

Dataset 1

Supplementary Information

## Data Availability

The code to process the MedAESQA and evaluate the system can be found at GitHub (https://github.com/deepaknlp/MedAESQA).
