# Supplementary material for: A Dataset of Medical Questions Paired with Automatically Generated Answers and Evidence-supported References
Source: Sci Data. 2025 Jun 19;12:1035. doi: 10.1038/s41597-025-05233-z (PMC12179289; doi:10.1038/s41597-025-05233-z)
Supplement: Supplementary file 2 — Supplementary Information [file 41597_2025_5233_MOESM2_ESM.pdf]

Medical Question Answering Dataset Generation Guidelines

These guidelines were used to generate the MedAESQA collection and may support an expansion of the existing collection or generation of similar datasets, e.g., addressing information needs of clinicians or focusing on a specific clinical area. The tasks addressed by MedAESQA are: 1) question understanding; 2) answer generation; 3) finding relevant documents for answer support; and 4) providing evidence support for each atomic statement in the answer. The dataset can be used both to develop and test various approaches to the tasks.

Question Selection

To develop and test QA systems on realistic questions, questions must be drawn from actual user inquiries. For MedAESQA, the questions were drawn from the requests submitted to MedlinePlus, ensuring the dataset captures real-world concerns rather than only curated academic prompts. In the absence of logs and requests submitted to customer services, questions posted to the online communities that allow using posts for research may be used, e.g., we used Yahoo! Answers in the question summarization collection. Independently of the source, the novelty and potential impact of the questions must be verified. For example, if the question – answer pairs exist in other annotated collections, the question may be included if the answer has changed due to new evidence or additional annotations are needed for the tasks for which the collection is created. Simple questions about common diseases should be avoided. While traditionally the frequently asked questions were used, the advances in technology call for expanding the pull with more complex questions, preferably with answers that require pulling together multiple sources of evidence and reasoning. For example, “what is hypertension” should not be included, but a less frequently asked question “are there drug interaction between doxylamine succinate and morphine, oxycodone, or lorazipam?” is a good candidate.

Evidence-supported Answer Generation

The workflow envisions that the questions are answered by clinicians with good knowledge of the current understanding of the topic. The clinician must provide a complete answer in professional register. The answer length must be between 30 and 300 words (up to 15 sentences). Each statement in the answer must be supported by at least one, and up to three PubMed references. The order in which the references are found may vary. For example, a clinician may choose to search PubMed first for the latest strongest evidence on the topic and then write the answer supporting the statements with evidence. Alternatively, clinicians may answer the question first and then either search themselves or engage a medical librarian to find PubMed documents supporting their statements. The documents should be selected/ranked using the EBP guidelines (i.e., the strongest evidence from the sources with

known rigorous peer-review process is preferred
https://www.ncbi.nlm.nih.gov/books/NBK470182/
https://www.merckmanuals.com/professional/special-subjects/clinical-decision-making/evidence-based-medicine-and-clinical-guidelines#Evidence-Based-Medicine_v1123723
). To generate reference (best-possible) answers, the annotators may follow either of the two paths. Note, while the documents are restricted to publications indexed in PubMed, any information retrieval system may be used to find relevant documents.

Use of LLMs at any stages of the process is prohibited. For each answer statement and each reference, the statements (parts of the abstract) that support the answer assertion must be linked to it.

The generated professional-level answers then need to be adapted to the patients' health literacy level.

The general requirement for the best possible answers is that the answer is:

1. Full, yet as short as possible (and not exceeding max allowed wordcount).
2. Each fact is supported by up to 3 PubMed citations with the strongest level and most up-to-date evidence possible.
    - The referenced documents must explicitly support the answer (no inference is allowed). The part of the document that supports the statement should be provided as a text snippet linked to the answer assertion (see example below).
3. Does not contain unnecessary information, such as general information on the topic or recommendations to see a doctor – the patient has already contacted the provider, or the provider is asking the question.
4. Clearly indicates the lack of consensus on the topic, if exists, and provides pros and cons for the alternatives.
5. Specifically addresses the patient's demographics, if present in the question.

The answer should first be generated at the professional level and then adapted to the patient register. Both answers should be provided in the deliverables. In addition, to facilitate automated evaluation of the LLM-generated answers, the annotators must provide a list of atomic statements that must be present in the answer (see Sample answer).
Having three answers to each question (6 total) would be ideal but not required. The maximal answer length allowed is 15 sentences, about 200-300 words. The minimal length is 3 sentences, about 30-100 words. Each sentence preferably should contain one fact, but if this is not possible, each atomic fact should be supported by an inline reference. The references should be provided as PMIDs in square brackets (a comma-separated list either after the statement of the fact, or after the sentence, but before the punctuation mark). Square brackets should not be used elsewhere in the text.

Each answer should be well-organized, in English, using complete sentences. Do not use any formatting (such as bulleted points, tables, bold-face type, etc.) to organize your answer.

The information needs (clinical tasks / intents) of the question fall into several categories: supporting decisions about treatments, finding the best diagnostic tests, differential diagnosis, adverse drug reactions, lifestyle changes, disease etiology and prognosis. For most of these tasks, EBP provides clear guidance on the expected answers. The annotators should follow these guidelines to provide the full answer.

Assumptions:
if patients are asking for treatments or diagnostic test, there is no need to provide information about the disease – they already know about it.
If the patient is asking about drug effects and no other information is provided, assume they are asking about side-effects.

Sample answer:

Topic: iron and ferritin levels in COVID-19
Question: why is transferrin and iron low in covid patients but ferritin high?
Narrative: The patient is interested in the link between iron and infection, the role iron plays in infection and the implications for COVID-19 course.

Sample Answer (professional level): During infections, a battle for iron takes place between the human host and the invading pathogens [34389110]. Lymphocytes need iron to mount an effective cellular and humoral response [34389110]. Viruses depend on iron to replicate within living host cells [31585922]. During the acute phase of infection, blood levels of iron decrease [37932342]. Ferritin levels are high [34883281]. Elevated serum ferritin is associated with increased mortality [34048587, 32681497]. As a major iron storage protein, ferritin is essential to iron homeostasis and is involved in a wide range of physiologic and pathologic processes [18835072]. The inflammation cascade and poor prognosis of COVID-19 may be attributed to high ferritin levels [34924800]. Iron depletion therapy was proposed as a novel therapeutic approach in the COVID-19 pandemic [32681497].

Atomic statements:
Lymphocytes and viruses compete for iron.
lymphocytes need iron for cellular and humoral response.
Viruses need iron to replicate.
Infection lowers iron levels in the blood.
High ferritin indicates response to inflammation.
Ferritin is maintaining the body's iron level.
High ferritin levels are linked to poor outcomes.
Iron depletion therapy showed anti-viral and anti-fibrotic activity in the COVID-19 pandemic.

Sample Answer (patient level): During infections, a battle for iron takes place between the human body and the invading viruses [34389110]. The immune system cells need iron to defend the body against the infection [34389110]. The virus needs iron to reproduce [31585922]. Iron balance is disrupted by the infection [37932342] and ferritin levels are high [34883281], which

signals the disease is severe and may have unfavorable outcomes [34048587, 32681497]. Ferritin is maintaining the body’s iron level [18835072]. Some researchers believe that high levels of ferritin not only show the body struggles with infection, but that it might add to the severity of disease [34924800]. To help covid patients, the doctors may lower the ferritin levels that are too high using drugs that capture iron [32681497].

References:

34389110:
A_S1: During infections, a battle for iron takes place between the human host and the invading pathogens.
A_S2: Once primed by the contact with antigen presenting cells, lymphocytes need iron to sustain the metabolic burst required for mounting an effective cellular and humoral response.

31585922
A_S3: Viruses depend on iron in order to efficiently replicate within living host cells.

37932342
A_S4: Hypoferremia induced by increased hepcidin would reduce iron in the environment of extracellular pathogens, and the increased LCN2 would inhibit siderophores, resulting in the prevention of the pathogen's iron acquisition in each manner during the acute phase of bloodstream infection.

34883281
A_S4: Ferritin was initially described to accompany various acute infections, both viral and bacterial, indicating an acute response to inflammation.

34048587
A_S4: Elevated serum ferritin and IL-6 levels associated with increased mortality and with reduced mortality at ferritin levels <100 ng mL-1.

32681497
A_S4: Numerous studies have demonstrated the immunomodulatory effects of ferritin and its association with mortality and sustained inflammatory process.

18835072
A_S5: Ferritin, a major iron storage protein, is essential to iron homeostasis and is involved in a wide range of physiologic and pathologic processes.

34924800
A_S6: The inflammation cascade and poor prognosis of COVID-19 may be attributed to high ferritin levels.

32681497

A_S7: Iron chelation represents a pillar in the treatment of iron overload. In addition, it was proven to have an anti-viral and anti-fibrotic activity. Herein, we analyse the pathogenic role of ferritin and iron during SARS-CoV-2 infection and propose iron depletion therapy as a novel therapeutic approach in the COVID-19 pandemic.
